# Palatable Medicines

**Published:** 1860-03

**Authors:** 


*Palatable Medicines.*

In the *Semi-Monthly Medical News* for Dec. 1st, Dr. T. E. Jenkins has an article upon the above subject, from which we extract the following:

"The operation of sugar-coating pills is very simple, and may be practiced by any one. It is done thus: After the pills are finished, they should be rolled between the fingers, previously moistened with thick mucilage of gum-arabic, and while still wet, thrown together in a capsule or saucer containing a sufficiency of very finely powdered sugar, and rotated therewith until the surface is completely covered, after which they may be exposed to a gentle heat to dry them. If necessary, this process may be repeated in order to give a thicker coating." * * * "Powders may be mixed with aromatized sugar, in order to disguise their nauseous qualities, and every practitioner should be provided with a few bottles of sugar aromatized with orange, lemon, peppermint, or other agreeable volatile oil, so as to have always at hand the means of rendering less disagreeable the chartulæ for his little patients." * * * "A very elegant *bonbon* may be made by incorporating powdered drugs, or even medicinal extracts, with pulverized aromatized sugar and white of egg, in the manner in which confectioners make their 'icing' for cakes, and dropping on a tile from the truncated end of a paper cone portions of the mixture suitable for a dose, they soon dry when exposed to the air, and will be eagerly sought after by children."

